# First-Principles Study on the High Spin-Polarized Ferromagnetic Semiconductor of Vanadium-Nitride Monolayer and Its Heterostructures

**DOI:** 10.3390/molecules30102156

**Published:** 2025-05-14

**Authors:** Guiyuan Hua, Xuming Wu, Xujin Ge, Tianhang Zhou, Zhibin Shao

**Affiliations:** 1 Basic Medical College, Binzhou Medical University, Yantai 264003, China; 2 College of Physics Science and Technology, Lingnan Normal University, Zhanjiang 524048, China; wuxm@lingnan.edu.cn; 3 School of Physics and Electric Engineering, Anyang Normal University, Anyang 455000, China; gexujin@aynu.edu.cn; 4 College of Carbon Neutrality Future Technology, China University of Petroleum (Beijing), Beijing 102249, China; zhouth@cup.edu.cn; 5 Physics Laboratory, Industrial Training Center, Shenzhen Polytechnic University, Shenzhen 518055, China

**Keywords:** monolayer h-VN, spin-gapless state, quantum anomalous Hall effect, high Curie temperature

## Abstract

The newly discovered 2D spin-gapless magnetic materials, which provide new opportunities for combining spin polarization and the quantum anomalous Hall effect, provide a new method for the design and application of memory and nanoscale devices. However, a low Curie temperature (*T_C_*) is a common limitation in most 2D ferromagnetic materials, and research on the topological properties of nontrivial 2D spin-gapless materials is still limited. We predict a novel spin-gapless semiconductor of monolayer h-VN, which has a high Curie temperature (~543 K), 100% spin polarization, and nontrivial topological properties. A nontrivial band gap is opened in the spin-gapless state when considering the spin–orbit coupling (SOC); it can increase with the intensity of spin–orbit coupling and the band gap increases linearly with SOC. By calculating the Chern number and edge states, we find that when the SOC strength is less than 250%, the monolayer h-VN is a quantum anomalous Hall insulator with a Chern number *C* = 1. In addition, the monolayer h-VN still belongs to the quantum anomalous Hall insulators with its tensile strain. Interestingly, the quantum anomalous Hall effect with a non-zero Chern number can be maintained when using h-BN as the substrate, making the designed structure more suitable for experimental implementation. Our results provide an ideal candidate material for achieving the QAHE at a high Curie temperature.

## 1. Introduction

In 1980, Klaus von Klitzing made a groundbreaking discovery known as the quantum Hall effect (QHE) while conducting experimental research on the electrical transport properties of two-dimensional (2D) electron systems [1]. Moreover, the non-dissipative chiral edge state is insensitive to the size and impurities of the sample, so it has potential applications in ultrafast transportation and ultralow energy quantum devices. However, the application of QHE is greatly limited by its realization in low temperatures and external magnetic fields. Haldane theoretically studied the two-dimensional hexagonal honeycomb crystal structure and found that the quantum Hall effect can occur without an external magnetic field, which is referred to as the quantum anomalous Hall effect (QAHE) [2,3]; this opened up exciting new possibilities for advancements in quantum physics and materials science. The QAH insulator, also known as the Chern insulator, can be obtained by introducing magnetism into a topological insulator (TI), which breaks time-reversal symmetry (TRS) and opens the band gap by considering SOC, resulting in a nontrivial state with the non-zero Chern number (*C*) [4]. The QAHE has attracted substantial theoretical attention in recent years [5,6,7,8], but little progress has been made in experiments. It was not until 2013 that the QAHE was first experimentally observed by Xue et al. in five quintuple-layer-thick Cr-doped (Bi, Sb)_2_Te_3_ films [9]. However, the ferromagnetism in the Cr-doped (Bi, Sb)_2_Te_3_ film system is highly disordered, resulting in an extremely low observation temperature of 30 mK [10,11]. Subsequently, the system comprising (Zn,Cr)Te/(Bi,Sb)_2_Te_3_/(Zn,Cr)Te, which incorporates the ferromagnetic insulator (Zn,Cr)Te and the topological insulator (Bi,Sb)_2_Te_3_, is designed to enhance the temperature of the quantum anomalous Hall effect (QAHE) to 100 mK [12]. Recently, Zhang et al. observed the zero-field QAHE in five septuple layers with the ferromagnetic topological insulator MnBi_2_Te_4_ and an ABC-trilayer graphene/hexagonal boron nitride (h-BN) moiré superlattice, with the observed temperatures reaching up to 1.4 K and 1.5 K, respectively [13,14]. Although the QAHE has been observed experimentally with a significant increase in temperature, there are still huge difficulties in its application in electronic devices due to its extremely low observation temperature. Therefore, it remains crucial to search for intrinsic QAHE materials with a high *T_C_* and high spin polarization.

Two-dimensional materials [15,16,17,18] have attracted great interest in academia and industry since graphene [19] was successfully exfoliated and revealed as an outstanding material with extraordinary mechanical stability [20], excellent optical transparency [21], exceptional electrical and superconductor ferromagnetic properties [22,23], a novel quantum hall effect [24], etc. So far, many 2D materials such as h-BN [25], silicene [26,27], black phosphorus [28,29,30,31], phosphorene [32,33], germanene [26,34], CrI_3_ [35,36], CrTe_2_ [37], ZrTe_2_ [38], Cr_2_Ge_2_Te_6_ [39], MXenes [40,41,42], transition metal dichalcogenides (TMDs) [43,44], transition metal nitride (TMNs) [45,46], transition metal carbides (TMCs) [47,48], etc., have been synthesized successfully in experiments and their novel potential applications in electronic devices have been investigated. These 2D magnetic materials feature inherent layered structures, superior crystallinity, and favorable coupling with suitably selected or designed substrates. Their pronounced sensitivity to environmental factors, such as gate bias, molecular adsorption, and interfacing materials, allows magnetism to be manipulated electrically, unlocking new avenues for integrating these magnets into spintronic systems and memory-based devices [44]. However, only a few 2D magnetic materials have been synthesized [44,48], and the ferromagnetism in monolayer structures has motivated the search for new materials with intrinsic spin interactions. In recent years, intrinsic magnetic vanadium nitride nanolayers (1–20 monolayers) with a (111) orientation have been experimentally obtained on MgO and Pt (111) substrates [49], which have made them a potential candidate for spintronic device applications. Recently, it has been reported that the h-VN monolayer is a ferromagnetic half-metallic material with a high Curie temperature (768 K) [50]. In addition, the 2H-VS2/h-VN and WS_2_/h-VN heterostructure generates considerable valley splitting [51,52]. However, there are few reports concerning the different structural stacking patterns, the biaxial strain, and the different SOC strengths’ influence on the topological properties and magnetism of the VN monolayer and its heterostructure. In the present work, by employing first-principles calculations, we conduct a systematic investigation of the electronic, magnetic, and topological properties of the 2D hexagonal vanadium nitride (h-VN). Furthermore, we add a substrate of BN to h-VN and change the stacking pattern, which is used to explore the robustness of the topological properties for the heterostructure. Our results show that the monolayer h-VN could be potentially exfoliated from the bulk VN crystal, and exhibits a spin-gapless semiconductor (SGS) with an intrinsic ferromagnetic nature, 100% spin polarization, and a high Curie temperature (543 K). When the SOC is considered, a global gap is opened at the Fermi level of the electronic band, and the band gap increases with the enhanced SOC intensity factor *λ_soc_*. To validate the practical application of h-VN in spintronic devices, the stability, electronic, and topological properties of eight different configurations, including h-VN/h-BN heterojunctions and h-BN/h-VN/h-BN quantum wells, are carried out.

## 2. Results and Discussion

**Figure 1:**
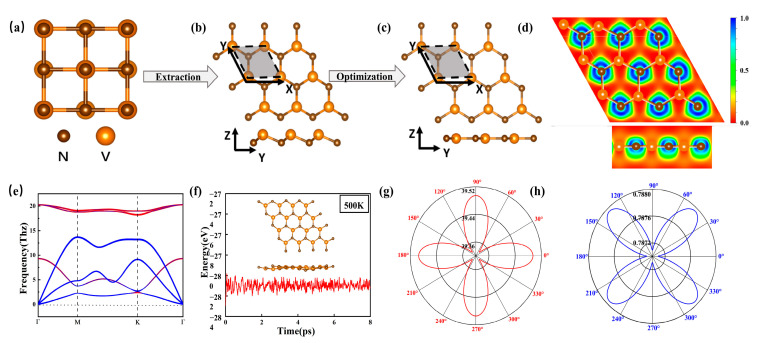
(**a**) The structure of bulk VN. (**b**,**c**) The top and side views of 2D VN after cutting along the [111] plane and the optimized structure. (**d**) The electron localization function (ELF) of monolayer VN. (**e**) The phonon spectrum of monolayer VN, the acoustic modes (blue curves) are primarily attributed to V atoms, and the optical modes (red curves) are mainly inhabited by N atoms. (**f**) The potential energy of VN after the 8 ps AIMD simulations at 500 K. The insets show the snapshot of the VN structure. (**g**) Young’s modulus and (**h**) the Poisson ration of monolayer VN.

Figure 1a shows that the bulk VN has a typical NaCl structure with an $Fm\bar{3}m$ space group, and that the V-N bond length and lattice constant of the bulk VN are 2.062 Å and 4.125 Å, respectively. The initial structure of the 2D two-atomic-layers-thick h-VN film (Figure 1b) can be theoretically exfoliated from the bulk VN along the (111) orientation. After the structural relaxation, the h-VN film transforms into a flat atomic monolayer, which is similar to monolayer CrN [50]. The lattice parameter and bond length of the V-N for monolayer VN are 3.244 Å and 1.873 Å, respectively. The 2D flat h-VN exhibits crystallographic symmetry corresponding to the space group $P\bar{6}m2$ with the $D_{3h}$ point group. As shown in Figure 1c, each VN unit cell contains one V and one N atom with a coordination number 3, and the N atom and V atom occupy the A and B sites. In order to understand the bonding magnitude of h-VN, we calculate the cohesive energy ($E_{coh}$) per until cell of monolayer h-VN by the following formula [50,53]:

(1) $$E_{coh}=E_{VN}-{\;E}_{N}-E_{V}$$

where $E_{N}$ and $E_{V}$ are the energies of the isolated N and V atoms and $E_{VN}$ is the total energy of the VN unit cell. The result of $E_{coh}$ is 8.81 eV, which is larger than the SnGe (8.30 eV), InAs (7.85 eV), and InSn (7.11 eV), and is comparable to that of the SnSi (8.72 eV); this reveals that the monolayer h-VN will form a strongly bonded network. The Bader analysis [54] suggests that the electron charge transfer is 1.83 *e* from the V atom to the N atom. Figure 1d presents the electron localization function (ELF) [55] to elucidate the bonding nature, where the blue/red regions indicate electron accumulation/depletion. From Figure 1d of the ELF map, it can be seen that electrons are primarily localized around N atoms, while fewer electrons are found near V atoms. This suggests an ionic bonding character in the V-N bond of monolayer h-VN, which aligns with the results of the Bader charge analysis.

In order to investigate the structural stability of the monolayer h-VN, the formation ($E_{form}$) is obtained by using the expression:

(2) $$E_{form}=E_{VN}-\frac{1}{2}\mu_{N}-{\frac{1}{2}\mu }_{V}$$

where the $\mu_{N}$ and $\mu_{V}$ are the chemical potential of body-centered cubic (BCC) vanadium crystals and N_2_ molecules, respectively. The value of the formation energy for monolayer h-VN is −4.04 eV, which indicates that there is an exothermic reaction.

Figure 1e presents the phonon dispersion of monolayer h-VN. The lack of imaginary frequency throughout the BZ indicates the monolayer h-VN is dynamically stable. Additionally, the phonon dispersion with different color weights to represent the contributions of N and V atoms are shown in Figure 1e. It is obvious that the acoustic modes (blue curves) are primarily attributed to V atoms, and the optical modes (red curves) are mainly inhabited by N atoms. The thermodynamic stability of monolayer h-VN is confirmed by AIMD simulation. The results in Figure 1f show that the system is thermally stable at 500 K. The elastic deformation of the material significantly influences its electronic properties. We next calculate the elastic constants of monolayer h-VN based on the Born–Huang stability criteria, and the values of the four constants are $C_{11}={\;C}_{22}=103.8$ N/m, $C_{12}=81.7\;N/m$, and $C_{66}=11.0\;N/m$, respectively, which satisfy the mechanical stability criterion for 2D crystalline systems [56], i.e., $C_{11}>0$, $C_{66}>0$, $C_{11}C_{22}-C_{12}^{2}>0$. To describe the ability of monolayer h-VN to resist external forces, in-plane Young’s modulus *Y*$\left(\theta \right)=\frac{C_{11}^{2}-C_{12}^{2}}{C_{11}}$ and the Poisson ration $\nu \left(\theta \right)=\nu_{12}=\nu_{21}=C_{12}/C_{11}$ [57], and the result of the calculations are obtained in Figure 1g. From the results we can determine that the monolayer h-VN is isotropic, and the corresponding values of Young’s modulus and the Poisson ration are 39.50 N/m and 0.787, respectively. Moreover, the value of Young’s modulus is smaller than that of graphene (340 N/m) [20], h-BN (271 N/m) [58], and MoS_2_ (129 N/m) [59], suggesting that monolayer h-VN possesses better mechanical flexibility.

**Figure 2:**
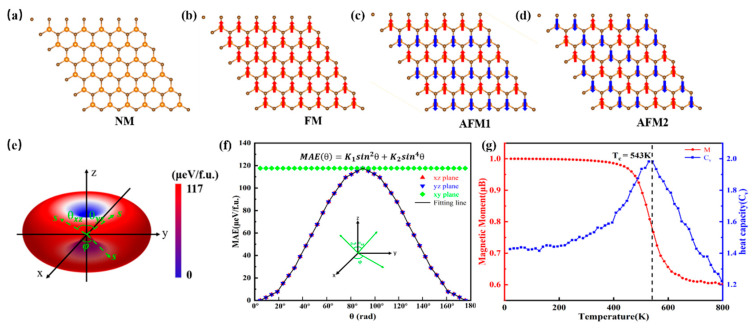
(**a**–**d**) The different magnetic configurations of VN. (**e**) The spatial angular dependence of the MAE of monolayer VN. (**f**) The MAE for monolayer VN with the polar angle *θ*. (**g**) The magnetic moment of the V atoms (b) and heat capacity as a function of the temperature for monolayer VN.

We conduct total energy spin-polarized calculations using 6 × 6 × 1 supercells for nonmagnetic (NM), ferromagnetic (FM), anti-ferromagnetic1 (AFM1), and anti-ferromagnetic2 (AFM2) phases for the VN monolayer to determine the magnetic ground state, as shown in Figure 2a–d. By comparison, the FM state is the most favorable state, while the NM, AFM1, and AFM2 states have energy differences (ΔE) of 380.71 meV, 160.67 meV, and 133.89 meV per unit cell with respect to the FM state, respectively. Remarkably, the h-VN monolayer shows a magnetic moment of 2 *μ*_B_ per unit cell and retains its magnetic properties during the simulation at 500 K, suggesting that the magnetic state of the h-VN monolayer remains stable at room temperature. The magnetic anisotropy energy (MAE) [60,61] is one key factor in achieving the long-range magnetic ordering for 2D materials. We calculate the energy considering the SOC interaction with magnetic moments along the (100), (010), (111), and (001) directions to explore the easy magnetization axis of monolayer h-VN. The calculated results of the calculation suggest that the easy magnetization axis is in the out-of-plane (001) direction with the lowest energy, which is lower than that along the (100), (010), and (111) directions by 117.6 *μ*eV/f.u., 117.6 *μ*eV/f.u., and 77.8 *μ*eV/f.u., respectively. Thus, we further discuss and calculate the MAE of 2D monolayer h-VN taking SOC into account. The MAE can be defined as the energy difference between Eθ and $E_{z}$, where Eθ and $E_{z}$ represent the total energy of the system when the magnetic moments are along the arbitrary orientation angle *θ* (*θ* ∈ [0°, 180°)) and the [001] direction, respectively. Figure 2e illustrates the angular dependence of the MAE for h-VN in the whole space; it can be observed that the MAE in the *xz* and *yz* planes strongly depends on the magnetization direction, while it is isotropic in the *xy* plane. We next fit the MAE equation which is dependent on the in-plane pole angle *φ* (*φ* ∈ [0, 360)) and the out-of-plane azimuthal angle *θ* through

(3) $$MAE\left(\theta \right)=K_{1}{sin}^{2}\theta +K_{2}{sin}^{4}\theta$$

where $K_{1}$ and $K_{2}$ denote the anisotropy coefficients, and *θ* is the arbitrary orientation angle measured with respect to the easy axis. An effective value of $K_{1}$ = 117 *μ*eV and $K_{2}$ = −0.15 *μ*eV can be obtained from Figure 2f. The anisotropy coefficient $K_{1}$ is positive and indicates that the easy magnetization direction of the system will prefer the out-of-plane easy axis. Compared to the $K_{1}$, the $K_{2}$ can be ignored. Moreover, the large MAE makes h-VN a potential candidate material for magnetoelectronic applications.

Next, we estimate the *T_c_* of monolayer h-VN by a Monte Carlo simulation based on the classical Heisenberg model, where the Hamiltonian can be given by:

(4) $$H=-\sum_{<i,j>}J_{1}\vec{S_{i}}\cdot \vec{S_{j}}-\sum_{<i,k>}J_{2}\vec{S_{i}}\cdot \vec{S_{k}}-\;A\sum_{i}{{(s}_{i}^{z})}^{2}$$

where $J_{1}$($J_{2}$) represent the nearest-neighbor (NN) (the next nearest-neighbor (NNN)) exchange parameters, respectively. The $\vec{S_{i}}$ is the spin vector of the V atom at site *i*, A is the magnetic anisotropy coefficient, and $s_{i}^{z}$ denotes the *z*-component of the spin vector. Therefore, we consider these neighbor couplings, and then the values of $J_{1}$, $J_{2}$, and A can be expressed approximately as:

(5) $$E_{FM}=\;E_{0}-36\times \left(3J_{1}\left|{S|}^{2}+3J_{2}\right|{S|}^{2}\right)-A|{S|}^{2}$$

(6) $$E_{AFM1}=\;E_{0}-36\times \left(-J_{1}\left|{S|}^{2}-J_{2}\right|{S|}^{2}\right)-A|{S|}^{2}$$

(7) $$E_{AFM2}=\;E_{0}-36\times \left(-J_{1}\left|{S|}^{2}+J_{2}\right|{S|}^{2}\right)-A|{S|}^{2}$$

(8) $$A=\frac{\left[E_{100}-E_{001}\right]}{|{S|}^{2}}$$

where $E_{0}$, $E_{FM}$, $E_{AFM1}$, and $E_{AFM2}$ represent the total energy of the NM, FM, AFM1, and AFM2 configurations from the DFT calculations. The values of $J_{1}$, $J_{2}$, and A are 20.33 meV, −0.59 meV, and 1.63 meV. The Monte Carlo simulations are performed on a 32 × 32 × 1 supercell with periodic boundary conditions, where the spins at all sites are randomly flipped in each simulation, and 40,000 sweeps are used to thermalize the system in order to achieve equilibrium sufficiently. Figure 2g illustrates the temperature dependence of the magnetic moment per unit cell, which begins to drop dramatically at 543 K, implying a transition from the ferromagnetic to paramagnetic phase. This value is much higher than that of the experimental ferromagnetic 2D materials, such as CrI_3_ (45 K) [35], Cr_2_Ge_2_Te_6_ (30 K) [39], and MnBi_2_Te_4_ thin films (25 K) [62]. The underlying reason for the high *T_c_* in h-VN can be attributed to the strong strength of the isotropic exchange interaction and the large value of MAE and the magnetic moment. The dxy and dx2-y2 states in the same spin channel primarily contribute to the PDOS of the V-3*d* orbital, which correspond to the possession of the high and positive values of the second term of the spin-flipping transition, resulting in the large exchange interaction, large MAE, and high *Tc*.

**Figure 3:**
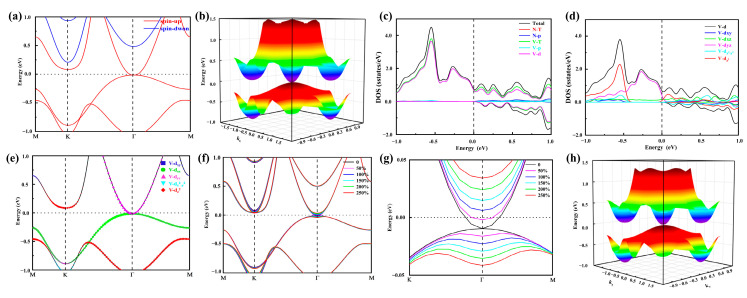
(**a**) The spin−polarized band structure; (**b**) the 3D band structure without SOC; (**c**) the total, N-total, N-*p*, V-total, N-*p*, and V-*d* PDOS; (**d**) the PDOS of the V-3*d* orbital; (**e**) the projected band structures in the spin-up channel; (**f**) the band structures with the intensities of SOC; (**g**) the partial enlarged view of the band structures with varying intensities of SOC at the high symmetry point *Γ*; (**h**) the 3D band structure with SOC for monolayer h-VN.

In Figure 3a, we show the band structure and projected density of states (PDOS) of monolayer h-VN in the most stable FM configuration without including the SOC effect. The spin-up and spin-down channels are displayed in different colors, respectively. Additionally, the spin-down channel has the semiconductor character with a ~3.1 eV band gap, while the spin-up channel exhibits an SGS feature with parabolic band dispersion at the high-symmetry *Г* point, which can also be viewed by the three-dimensional (3D) fully spin-polarized band structure in Figure 3b. The PDOS (Figure 3c) suggests that the *d* orbital of the V atom makes a major contribution to the spin-gapless point. We next analyze the PDOS and partial orbital-projected bands of the V-3*d* orbitals for the spin-up channel of monolayer h-VN. As shown in Figure 3d,e, we can determine that the spin-gapless point for the spin-up channel near the Fermi level arises primarily from the hybridization of the *d*_xz_ and *d*_yz_ states of the V atoms.

As a transition metal element, vanadium may be affected by SOC, and the form of the SOC effect is determined as follows:

(9) $$H_{SOC}\;\propto \;\sigma \cdot {\;B}_{eff}\propto \;\sigma \cdot \;\left(\nabla V\times p\right)=\lambda_{SOC}\nabla V\cdot \;\left(p\times \sigma \right)$$

where the σ, *p*, and *V* represent the vector of Pauli spin matrices, the momentum operator, and the Coulomb potential of the atomic core, respectively. The $\lambda_{SOC}$ is the parameter of SOC; we continue to explore the scaling factor $\lambda_{SOC}$ of the SOC term to investigate the influence of different strengths of SOC. The spin-polarized band structure and its partial enlarged view are shown in Figure 3f,g. We can clearly see that the band gap of monolayer h-VN increases with the increasing SOC intensity factor $\lambda_{SOC}$, ranging from 0 to 250%. Moreover, when the value of $\lambda_{SOC}$ reaches 100%, the SOC induces a ~ 23.83 meV opened gap in the spin-gapless states, which agrees with the 3D band structure in Figure 3h and leads to the nontrivial topological state, while the band gap is ~ 61.75 meV when the $\lambda_{SOC}$ reaches 250%. Interestingly, this case still maintains QAHE.

**Figure 4:**
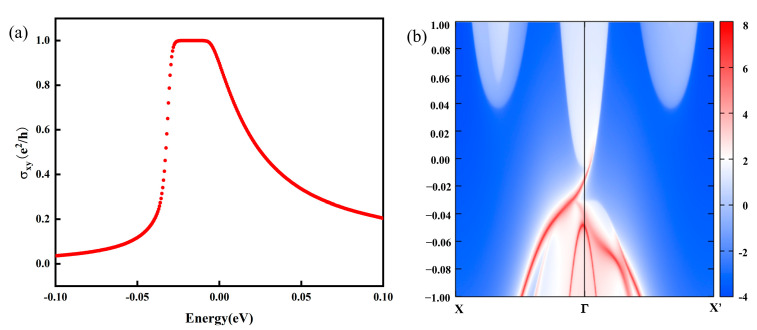
(**a**) The anomalous Hall conductance (AHC) *σ*_xy_ as a function of chemical potential, while the integer quantum conductance with terrace values occurs around the Fermi level. (**b**) The chiral edge states of monolayer h-VN.

The nontrivial topological electronic properties are further confirmed by the non-zero anomalous Hall conductivity ($\sigma_{xy}$) and Chern number (*C*), expressed by the formula below [63,64].

(10) $$\sigma_{xy}=Ce^{2}/h$$

(11) $$C=\frac{1}{2\pi }\int_{BZ}Ω\left(k\right)d^{2}k$$

(12) $$Ω\left(k\right)=\underset{n}{-2\sum f_{n}}\underset{m\neq n}{\sum }Im\frac{\left\langle \Psi_{n}(k)\left|\nu_{x}\right|\Psi_{m}(k)\right\rangle \left\langle \Psi_{m}(k)\left|\nu_{y}\right|\Psi_{n}(k)\right\rangle \hbar^{2}}{{\left(E_{m}{-E}_{n}\right)}^{2}}$$

where *e*, *h*, $Ω\left(k\right)$, $f_{n}$, $\Psi_{n}(k)$, and $E_{n}$ are the electronic charge, Planck constant, Berry curvature, Fermi–Dirac distribution function, Bloch wave function, and eigenvalue, respectively. The $\nu_{x/y}=\frac{1}{\hbar }\frac{𝜕H}{{\partial k}_{x/y}}$ is the velocity operator. According to Equations (6) and (7) and through integrating the Berry curvature in the vicinity of the quadratic band, a value of 2π is obtained for the Berry curvature $Ω\left(k\right)$, indicating that the quadratic crossing point has a topological charge of 1 and an AHC of e^2^/h, corresponding to the non-zero Chern number with the integerized value of 1 and the quantized Hall conductance. Herein, all the values of the Chern number C are maintained at 1 when $\lambda_{SOC}$ increases from 1% to 250%, meaning that h-VN has a robust nontrivial topological state and the Chern number is insensitive to SOC strength. As expected, the anomalous Hall conductivity correspondingly shows a quantized Hall plateau $\sigma_{xy}(1\times e^{2}/h)$ around the Fermi level, as shown in Figure 4a. An iterative calculation of Green’s function for a semi-infinite VN steet is carried out by using the effective principal layer concept. The local density of states (LDOS) at the edge, as a function of energy and momentum, can be derived from the imaginary part of the surface Green’s function. As expected in Figure 4b, the surface states in the VN monolayer occur, as indicated by the highest red color intensity at the *Γ* point. We see that an edge state connects the valence and conduction bands across the insulating gap, which confirms the characteristics of QAHE and that this system is a QAH insulator. Such results are consistent with the Chern number *C* = 1.

Applying strain to the crystal structure of the materials is also an effective method for regulating the electronic structure, as strain can cause changes in the bond lengths between material atoms, which may affect the orbital hybridization and band structure of the system. To gain a deeper understanding of the impact of biaxial strain on the stability, magnetism, electronic structure, and topological properties of the h-VN monolayer, we apply in-plane biaxial strain ranging from −5% to 5% to examine the properties of the monolayer h-VN. The strain is defined as $\varepsilon =(c={\;c}_{0})/c_{0}\times 100\%$, where the *c* and *c*_0_ are the lattice parameters of monolayer h-VN for strained and unstrained systems, and the ε<0 and ε>0 represent the compressive and tensile strain, respectively. To confirm the dynamic stability of monolayer h-VN under strain, as shown in Figure S1, based on the results of the phonon spectrum structure, it can be seen that there are imaginary frequencies in the phonon spectrum of the compressed strain monolayer h-VN, while the whole Brillouin zone of the tensile strain monolayer h-VN has no imaginary frequencies. The value of the elastic constants $C_{11}$, $C_{22}$, $C_{12}$, and $C_{66}$; the cohesive energy; and the formation energy of monolayer h-VN after biaxial strain are shown in Table S1. The results show that the $C_{66}$ under compression strain with −5%, −4%, and −3% is less than zero, indicating the three biaxial strains do not satisfy the mechanical stability criterion.

Next, we calculate the magnetic properties, band structures, and topological properties of monolayer h-VN with biaxial strain. Across the biaxial strain range (−5% to 5%), the energies of the FM states of the monolayer h-VN are lower than those of the two AFM states, which indicates that the ferromagnetic properties are robust for the monolayer h-VN. Moreover, the total magnetic moment for a monolayer h-VN unit cell remains at 2 *μ*_B_, even after applying strain, indicating that the magnetic moment of the V atom in the monolayer h-VN does not change within the biaxial strain range of −5% to +5%. In addition, the Curie temperature of monolayer h-VN with biaxial strain is calculated, and the results of the calculation for the NN and NNN exchange coupling parameters $J_{1}$ and $J_{2}$ are shown in Table S1. As shown in Figure S2, the strain has little effect on its Curie temperature. As shown in Figure S3, monolayer h-VN exhibits metallic properties under compressive strain ranging from −5% to −2%, while it remains an SGS under −1% compressive strain and tensile strain ranging from 1% to 5%, meaning the SGS characteristics include robustness to the positive tensile strains. Therefore, we next calculate the topological properties of monolayer h-VN under tensile strain, and the results of the calculation are shown in Figure S4. It can be seen that monolayer h-VN still maintains nontrivial topological properties with a Chern number of *C* = 1. The stability of the magnetic, electronic structure, and topological properties of monolayer h-VN under tensile strain greatly increases the possibility of its application in high-performance spin-electronic devices.

In order to demonstrate the practical applications in spintronic devices, we investigate the electronic properties of the h-BN heterojunctions with the hexagonal h-BN substrate. The h-BN substrate is a commonly used substrate in experimental studies [65,66] Since the optimized lattices of the h-VN and h-BN monolayers are 3.244Å and 2.51Å, respectively, in order to reduce the lattice mismatch, 2 × 2 h-VN and $\sqrt{7}$ × $\sqrt{7}$ h-BN are taken to construct heterojunctions and quantum wells; thus, a small lattice mismatch of 1.2% occurs in the system, which is smaller than the large lattice mismatches in some experimentally and theoretically explored research, such as SnSe_2_/MoS_2_ (20%) [67], GaSe/MoSe_2_ (13%) [68], and graphene/Cr_2_C [69].

**Figure 5:**
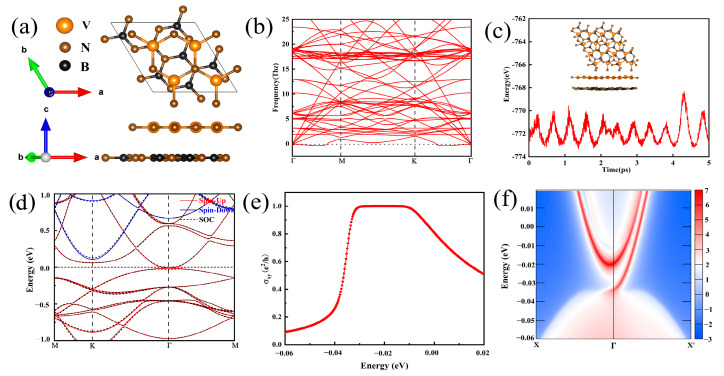
(**a**) The top and side views for the h-VN/h-BN heterostructure in the configuration I stacking pattern. (**b**) The phonon frequency. (**c**) The AIMD simulation at 500 K, where the inset is the structure of the heterostructure at the end of the AIMD simulation after 5 ps. (**d**) The band structure without and with SOC. (**e**) The anomalous Hall conductivity. (**f**) The chiral edge states of the h-VN/h-BN heterostructure on the configuration I stacking pattern.

We construct four possible configurations of h-VN/h-BN heterojunctions and h-BN/h-VN/h-BN quantum wells, named as (I, II, III, and IV) and (V, VI, VII, and VIII), by considering the high-symmetry positions, as shown in Figure 5a and Figures S5a–S11a, respectively. In the calculations, the DFT-D3 dispersion correction method is adopted for the van der Waals interactions between the VN and h-BN monolayers, as described in the computational methods. We calculate the phonon frequency and AIMD to determine the dynamic and thermodynamic stability of each heterojunction and quantum well. Obviously, as shown in Figure 5b and Figures S5b–S11b, the imaginary frequencies are absent in the whole BZ, which confirms the dynamic stability of the eight configurations. The AIMD calculations (Figure 5c and Figures S5c–S11c) for 5 ps (time step of 1 fs) at 350 K (300 K) suggest that the heterostructures and quantum wells are completely stable. All of the eight configurations can remain thermally stable above room temperature. The stability of heterostructures and quantum wells can also be predicted by the calculated binding energy $E_{b}$ [70], which can be expressed by:

(13) $$E_{b}=E_{H/Q}-E_{BN}-E_{VN}$$

where $E_{H/Q}$, $E_{BN}$, and $E_{VN}$ represent the total energy of the heterostructures/quantum wells, h-BN, and h-VN, respectively. A lower energy indicates the heterostructures/quantum wells are more stable. The calculation results are presented in Table 1, where the negative binding energy indicates the stable existence of the heterostructures/quantum wells. Configuration I/V (i.e., eclipsed stacking with V atom of VN over N atom of BN, simultaneously stacking with N atom of VN diagonal to V atom over NNN B atom of BN) is the most stable structure. To determine the mechanical stability of configuration I/V, we calculate the values for $C_{11}$, $C_{12}$, $C_{22}$, and $C_{44}$, and the results are 401.7/696.6, 136.6/127.5, 401.7/696.6, and 132.5/284.6 N/m, respectively, which satisfy the mechanical stability criterion, namely, $C_{11}>0$, $C_{66}>0$, $C_{11}C_{22}-C_{12}^{2}>0$.

We further calculate the interlayer distance *d* and the band gaps *∆*_NSOC_ (without SOC) and *∆*_SOC_ (with SOC). The results show that the interlayer distance *d* of configurations I/V is the smallest among the four heterostructures and quantum wells. The band structures of the heterostructures and quantum wells are shown in Figure 5d and Figures S5d–S11d. From those results, we know that heterostructures I, III, and IV are still SGSs, while heterostructure II and the four quantum wells are on longer SGSs with a small gap in the absence of SOC. When SOC is taken into account, the SGS points are opened for all eight configurations, whereas the band gaps of the heterostructures are smaller than those of the monolayer h-VN and larger than those of the quantum wells. Therefore, the h-BN substrate exerts minimal influence on the band structure of h-VN, suggesting that the interaction is predominantly of the van der Waals type. The opened band gaps at spin-gapless points by the SOC effect may lead to a nontrivial topological state. Then, we calculate the anomalous Hall conductivity (Figure 5e and Figures S5e–S11e) and edge state (Figure 5f and Figures S5f–S11f) of the eight configurations, indicating that both of the eight configurations can realize the QAHE with a Chern number *C* = 1. Thus, the heterostructures and quantum wells are a good way of experimentally synthesizing the h-VN monolayer while keeping its topological nontrivial properties and promising applications in electronic devices.

## 3. Computational Details

All first-principal calculations are performed using the Vienna Ab initio Simulation Package (VASP) [71]. The electron exchange-correlation potentials are treated with the generalized gradient approximation (GGA) by using the Perdew–Burke–Ernzerhof (PBE) functional [72,73]. The ion–electron interactions are described by the projector augmented wave (PAW) method [74]. A vacuum space of 15 Å in the *z*-direction is employed. The energy cutoff of the plane wave function is set above 450 eV, and the force and energy convergence thresholds are set to 0.01 eV/Å and 10^−6^ eV, respectively. Brillouin zone sampling use a 15 × 15 × 1 Γ-centered mesh [75]. The interlayer van der Waals (vdW) interactions are included by the DFT-D3 method [76]. The phonon frequency is obtained through the PHONOPY code based on the finite displacement method by integration with density functional perturbation theory (DFPT) [77]. The ab initio molecular dynamics (AIMD) simulation [78] is used to confirm the thermal stability. The Curie temperatures are obtained by Monte Carlo simulations. The maximally localized Wannier function calculations are implemented Wannier90 and WannierTools package [79,80,81].

## 4. Conclusions

In summary, based on the first-principles calculations, we systematically investigated the stability and electronic properties of monolayer h-VN. The thermal and dynamic stability of h-VN has been demonstrated by phonon dispersion calculations and AIMD simulations, respectively. The monolayer h-VN showed the intrinsic parabolic spin-gapless semiconducting ferromagnetic characteristics with 100% spin polarization in the absence of SOC. Monte Carlo simulation based on the classical Heisenberg model confirmed that the Curie temperature of monolayer h-VN is estimated to be as high as ~534 K. In the presence of the SOC, the monolayer h-VN became an intrinsic topological insulator with a Chern number *C* = 1 nontrivial band gap, and the band gap increased as the intensity of SOC increased from 0 to 250%, resulting in the QAHE. In addition, we found that the monolayer h-VN still exhibited the quantum anomalous Hall effect within a tensile strain range of 5%. To investigate the possibility of experimental implementation for the monolayer h-VN, we also constructed four kinds of heterojunctions and quantum wells using h-BN substrates and explored their stabilities, electronic structures, and topological properties. Our results provide ideal candidates for achieving high spin polarizability and QAHE in realistic systems, which could serve as a platform for dissipationless spintronic devices.

## Figures and Tables

**Table 1:** The binding energy *E*_b_, the interlayer distance *d* between the VN and BN monolayer, and the band gap ∆_NSOC_/*∆*_SOC_ without SOC for four differently stacking patterns.

Configuration	I/V	II/VI	III/VII	IV/VIII
*E*_b_ (eV)	−2.344	−2.343	−2.342	−2.342
	−4.249	−4.242	−3.050	−3.319
*d* (Å)	3.223	3.238	3.242	3.228
	3.203	3.269	3.294	3.310
*∆_N_*_SOC_ (meV)	0.00	1.12	0.00	0.00
	4.20	4.22	4.73	4.20
*∆*_SOC_ (meV)	23.51	23.49	23.53	23.50
	18.50	18.89	18.58	18.21

## Data Availability

The original contributions presented in this study are included in the article/Supplementary Materials. Further inquiries can be directed to the corresponding authors.

## Supplementary Materials

The following supporting information can be downloaded at: https://www.mdpi.com/article/10.3390/molecules30102156/s1, Figure S1: The phonon spectrum structures of h-VN with biaxial strain ranging from −5% to 5%; Figure S2: The magnetic moment of V atoms and heat capacity as a function of temperature for monolayer VN with biaxial strain ranging from −5% to 5%; Figure S3: The band structures of h-VN with biaxial strain ranging from −5% to 5%; Figure S4: The chiral edge states of h-VN with biaxial strain of −1% and ranging from 1% to 5%; Figure S5: (a) The top and side views for the h-VN/h-BN heterostructure in configuration Ⅱ stacking pattern. (b) The phonon frequency, (c) the AIMD simulation at 500 K, the inset is the structure of heterostructure at the end of the AIMD simulation after 5 ps, (d) the band structure without and with SOC, respectively, (e) the anomalous Hall conductivity, and (f) the chiral edge states of the h-VN/h-BN heterostructure with configuration Ⅱ stacking pattern; Figure S6: (a–f) are the same as Figure S5(a–f) but the h-VN/h-BN heterostructure with the configuration Ⅲ stacking pattern; Figure S7: (a–f) are the same as Figure S5(a–f) but the h-VN/h-BN heterostructure with the configuration Ⅳ stacking pattern; Figure S8: (a–f) are the same as Figure S5(a–f) but the h-VN/h-BN heterostructure with the configuration Ⅴ stacking pattern; Figure S9: (a–f) are the same as Figure S5(a–f) but the h-VN/h-BN heterostructure with the configuration Ⅵ stacking patter; Figure S10: (a–f) are the same as Figure S5(a–f) but the h-VN/h-BN heterostructure with the configuration Ⅶ stacking pattern; Figure S11: (a–f) are the same as Figure S5(a–f) but the h-VN/h-BN heterostructure with the configuration Ⅷ stacking pattern; Table S1: The elastic constant C, cohesive energy Ecoh, formation energy Ef, the nearest-neighbor and next nearest-neighbor exchange parameters J_1 and J_2, and Curie temperature TC of monolayer h-VN under the biaxial strain.
