# Supplementary material for: First-Principles Study on the High Spin-Polarized Ferromagnetic Semiconductor of Vanadium-Nitride Monolayer and Its Heterostructures
Source: Molecules. 2025 May 14;30(10):2156. doi: 10.3390/molecules30102156 (PMC12113942; doi:10.3390/molecules30102156)
Supplement: Supplementary file 1 [file molecules-30-02156-s001.zip › molecules-3514670-supplementary.pdf]

Supporting Information

First-Principles Study on the High Spin-polarized Ferromagnetic Semiconductor of Vanadium-Nitride Monolayer and its heterostructures

Guiyuan Hua [1,*], Xuming Wu [2], Xujin Ge [3], Tianhang Zhou [4], Zhibin Shao [5,*]

[1] Basic Medical College, Binzhou Medical University, Yantai 264003, China

[2] College of Physics Science and Technology, Lingnan Normal University, Zhanjiang 524048, China

[3] School of Physics and Electric Engineering, Anyang Normal University, Anyang 455000, China

[4] College of Carbon Neutrality Future Technology, China University of Petroleum (Beijing), Beijing 102249, China

[5] Physics Laboratory, Industrial Training Center, Shenzhen Polytechnic University, Shenzhen 518055, China

*Correspondence :huaguiyuan@bzmc.edu.cn(G.H.); zhibin_shao@szpu.edu.cn(Z.S.);

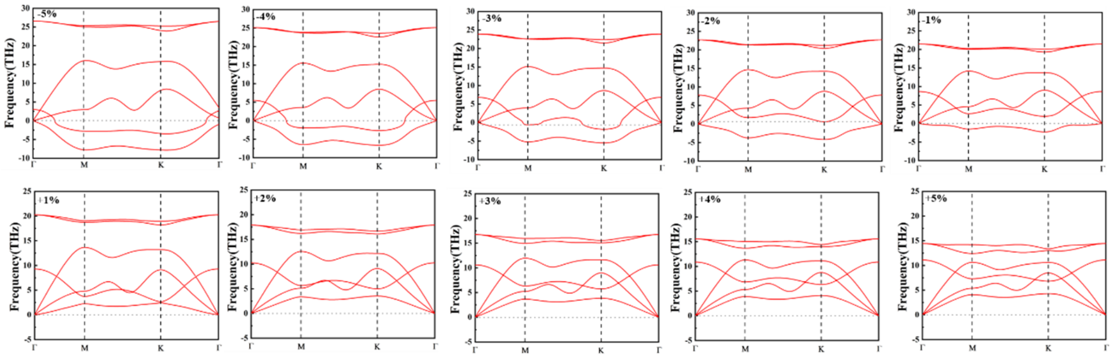

Figure S1. The phonon spectrum structures of h-VN with biaxial strain ranging from -5% to 5%.

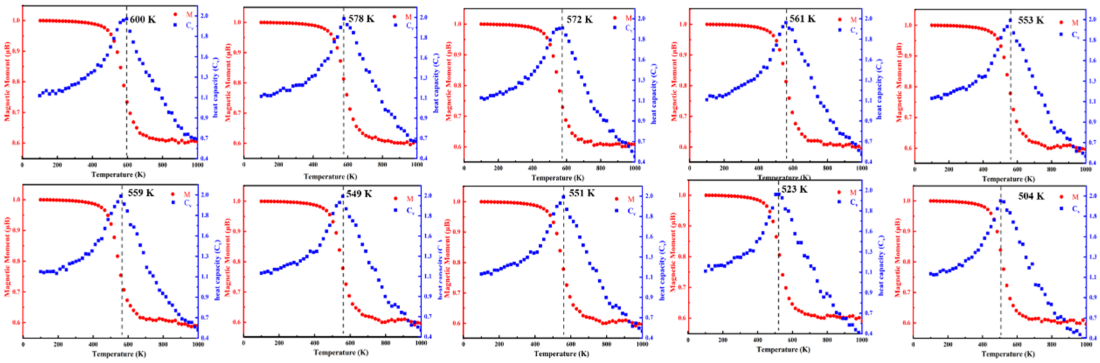

Figure S2. The magnetic moment of V atoms and heat capacity as a function of temperature for monolayer VN with biaxial strain ranging from -5% to 5%.

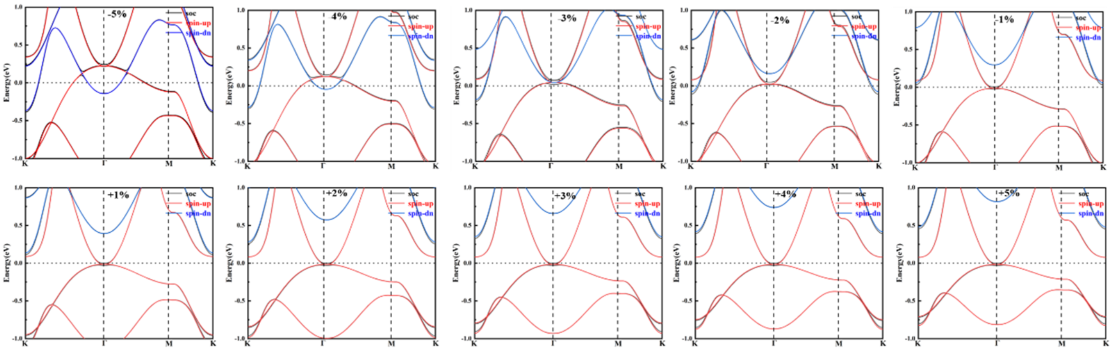

Figure S3. The band structures of h-VN with biaxial strain ranging from -5% to 5%.

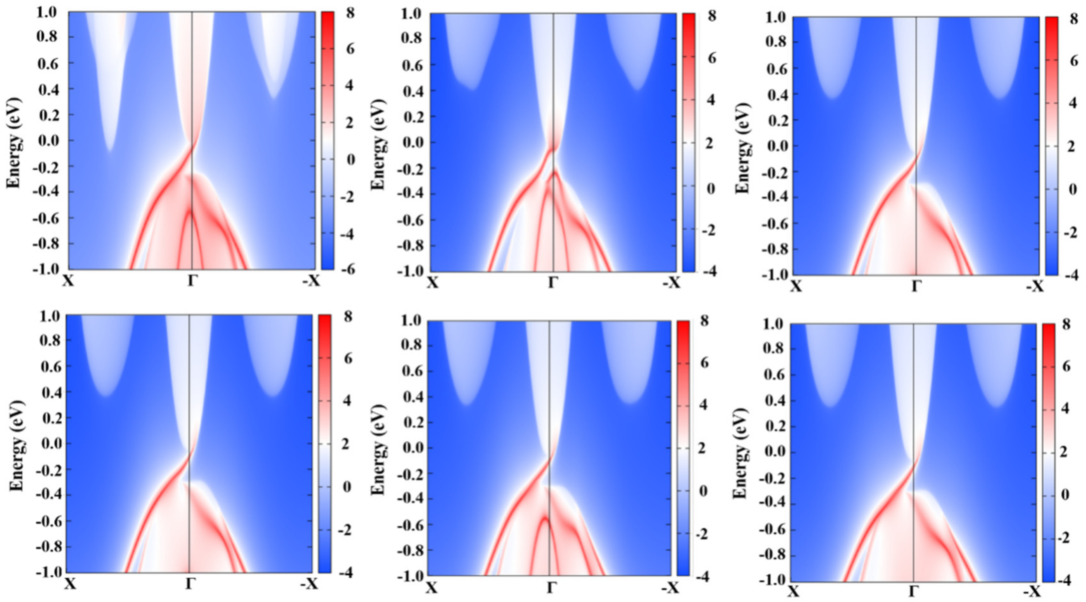

Figure S4. The chiral edge states of h-VN with biaxial strain of -1% and ranging from 1% to 5%.

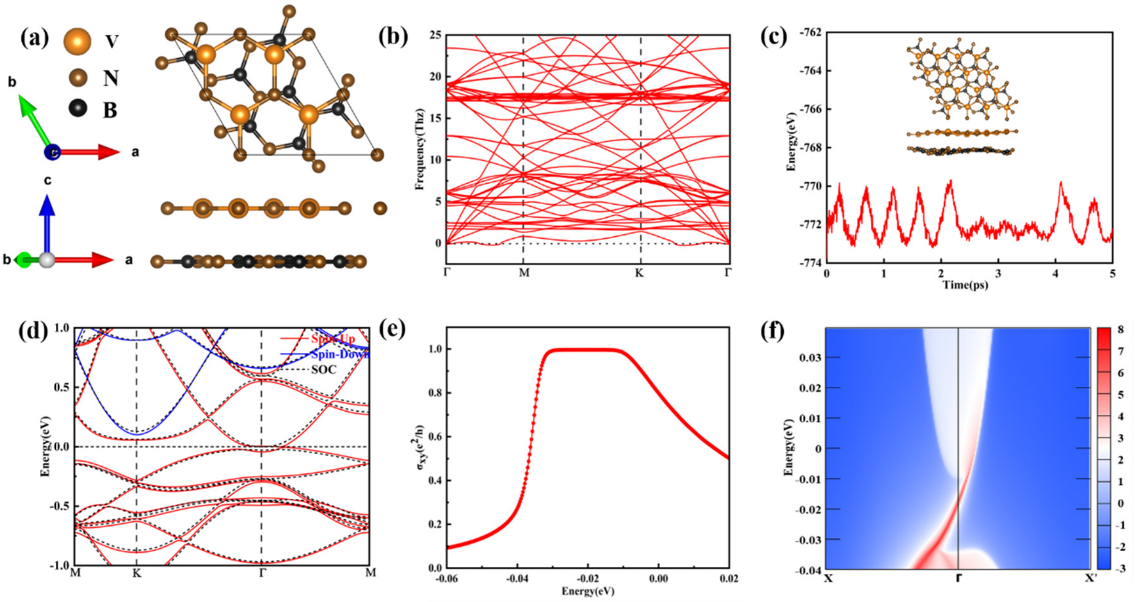

Figure S5. (a) The top and side views for the h-VN/h-BN heterostructure in configuration II stacking pattern. **(b)** The phonon frequency, **(c)** the AIMD simulation at 500 K, the inset is the structure of heterostructure at the end of the AIMD simulation after 5 ps, **(d)** the band structure without and with SOC, respectively, **(e)** the anomalous Hall conductivity, and **(f)** the chiral edge states of the h-VN/h-BN heterostructure with configuration II stacking pattern.

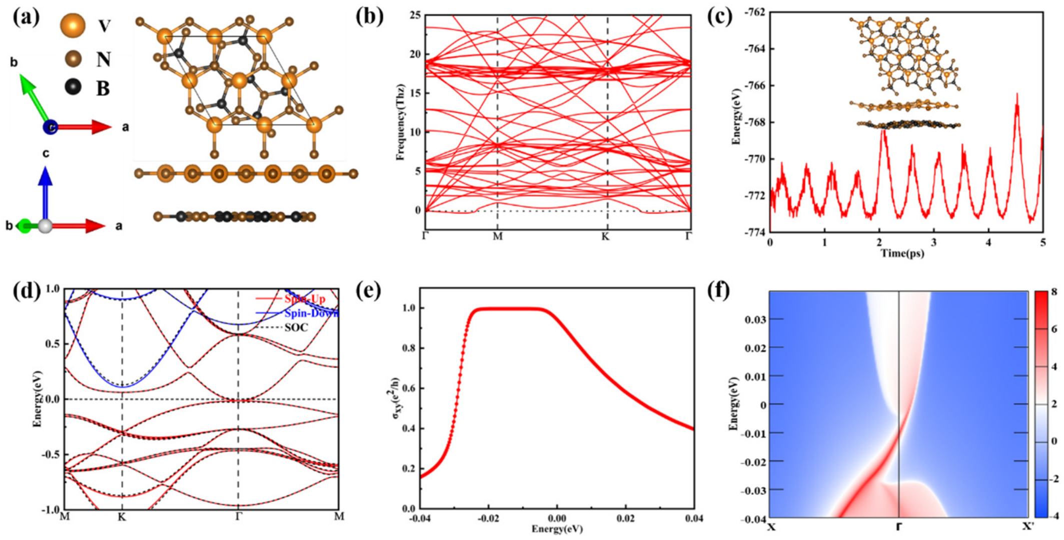

Figure S6. (a-f) are the same as Figure S5(a-f) but the h-VN/h-BN heterostructure with the configuration III stacking pattern.

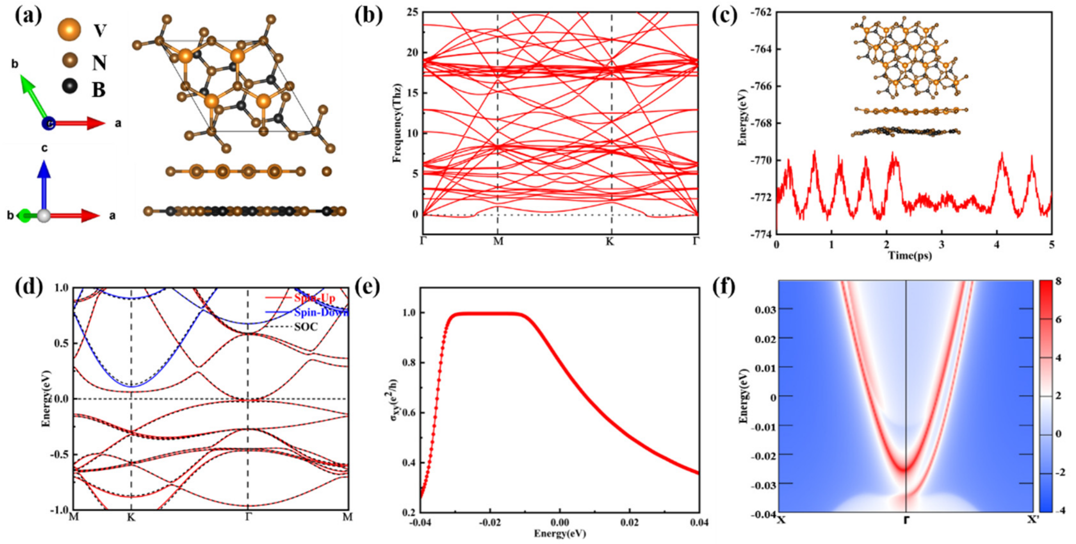

Figure S7. (a-f) are the same as Figure S5(a-f) but the h-VN/h-BN heterostructure with the configuration IV stacking pattern.

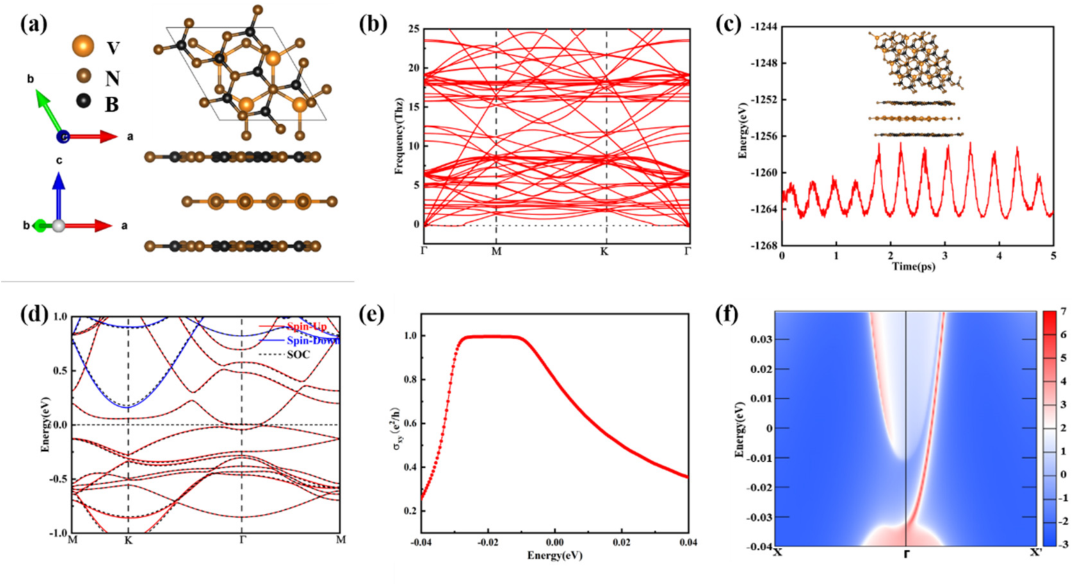

Figure S8. (a-f) are the same as Figure S5(a-f) but the h-VN/h-BN heterostructure with the configuration V stacking pattern.

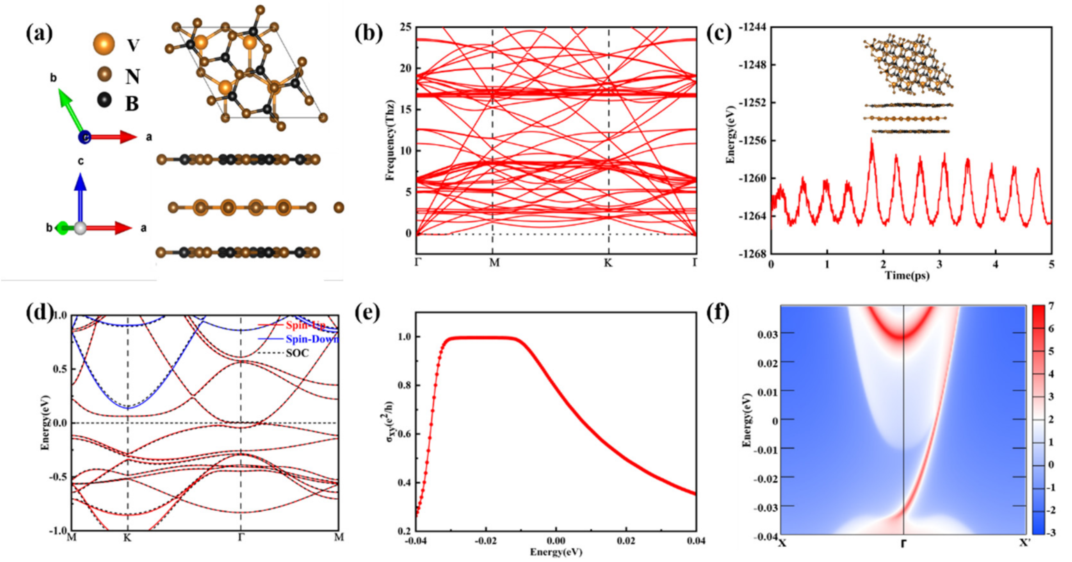

Figure S9. (a-f) are the same as Figure S5(a-f) but the h-VN/h-BN heterostructure with the configuration VI stacking pattern.

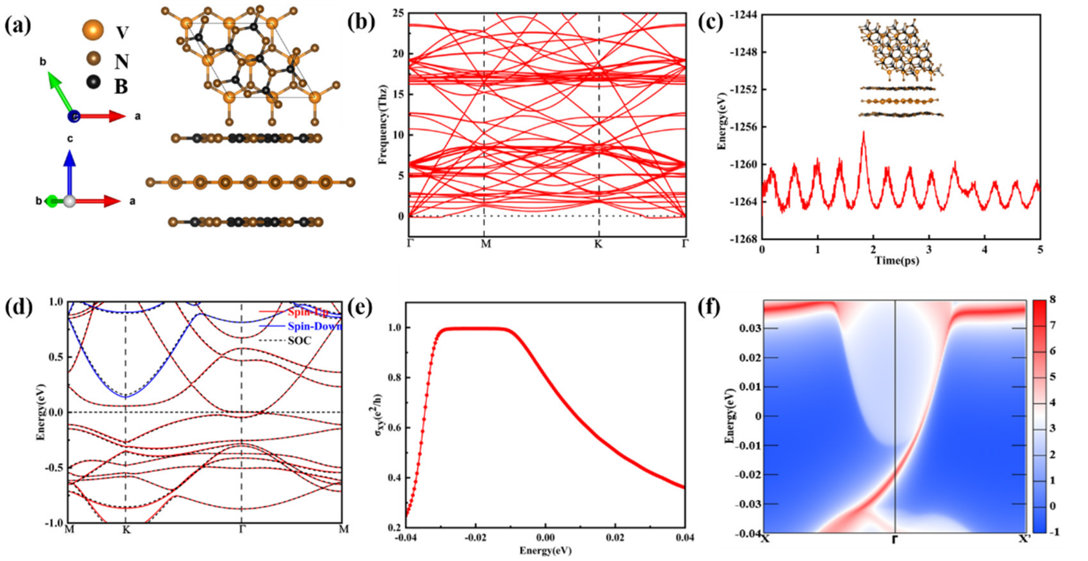

Figure S10. (a-f) are the same as Figure S5(a-f) but the h-VN/h-BN heterostructure with the configuration VII stacking pattern.

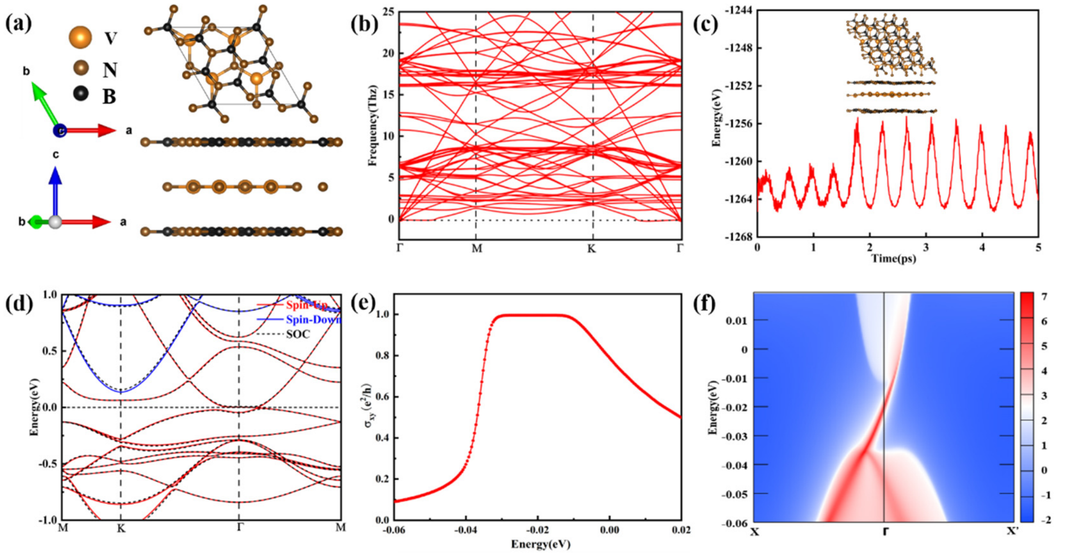

Figure S11. (a-f) are the same as Figure S5(a-f) but the h-VN/h-BN heterostructure

with the configuration VIII stacking pattern.

Table S1. The elastic constant *C*, cohesive energy Ecoh, formation energy E_f, the nearest-neighbor and next nearest-neighbor exchange parameters J_1 and J_2, and Curie temperature T_C of monolayer h-VN under the biaxial strain.

Strain %	C_{11} (N/m)	C_{12} (N/m)	C_{66} (N/m)	E_{coh} (eV/atom)	E_f (eV)	J_1 (meV)	J_2 (meV)	T_C (K)
-5	153.8	163.9	-5.1	4.21	-3.79	24.38	-0.61	600
-4	144.5	150.5	-3.0	4.27	-3.91	23.69	-0.66	578
-3	136.5	138.1	-0.8	4.31	-3.98	22.93	-0.67	572
-2	128.7	117.6	5.5	4.34	-4.03	22.15	-0.68	561
-1	120.4	104.6	7.9	4.38	-4.06	21.55	-0.68	553
0	112.0	92.7	9.7	4.41	-4.11	20.33	-0.59	543
1	95.6	71.9	11.9	4.34	-4.04	22.07	-0.67	559
2	84.2	63.1	12.2	4.32	-4.00	21.37	-0.66	549
3	79.3	55.3	12.0	4.29	-3.94	20.64	-0.65	551
4	76.9	52.7	12.1	4.26	-3.87	19.80	-0.64	523
5	71.4	48.6	11.5	4.21	-3.78	19.21	-0.62	504
